# How was apical growth regulated in the ancestral land plant? Insights from the development of non-seed plants

**DOI:** 10.1093/plphys/kiac313

**Published:** 2022-06-30

**Authors:** Jim P Fouracre, C Jill Harrison

**Affiliations:** School of Biological Sciences, University of Bristol, Bristol BS8 1TQ, UK; School of Biological Sciences, University of Bristol, Bristol BS8 1TQ, UK

## Abstract

Land plant life cycles are separated into distinct haploid gametophyte and diploid sporophyte stages. Indeterminate apical growth evolved independently in bryophyte (moss, liverwort, and hornwort) and fern gametophytes, and tracheophyte (vascular plant) sporophytes. The extent to which apical growth in tracheophytes co-opted conserved gametophytic gene networks, or exploited ancestral sporophytic networks, is a long-standing question in plant evolution. The recent phylogenetic confirmation of bryophytes and tracheophytes as sister groups has led to a reassessment of the nature of the ancestral land plant. Here, we review developmental genetic studies of apical regulators and speculate on their likely evolutionary history.

## Teaser

The combined results of recent developmental genetics and phylogenetics studies suggest that the ancestral sporophyte was more complex than previously thought.

## Introduction

The land plants we live among and depend on today look very different to the ancestral green alga that first colonized the land ∼470–515 million years ago (Steemans, 2000; Morris et al., 2018). In response to the new environmental challenges posed by life on land (e.g. desiccation, low water and nutrient availability, high UV radiation, and increased temperature variability), plants evolved a suite of morphological, physiological, and anatomical innovations. The ecological success of these adaptations were such that land plants transformed the planet, resulting in a huge diversity in form as different lineages evolved novel solutions to a changing world.

**Figure 1:**
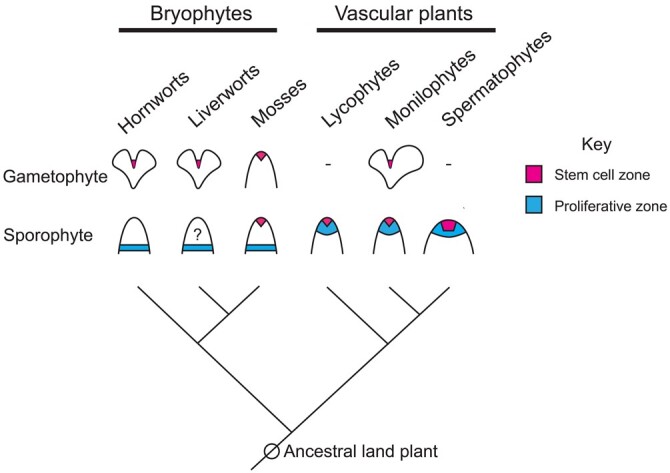
Phylogenetic relationships and representative meristem structure of land plants. Bryophyte gametophytes grow as thalli, leafy shoots or filaments, and meristems comprise a single apical cell. Monilophytes also produce thallus-type gametophytes with a notch meristem (in the case of *Ceratopteris* hermaphrodite gametophytes). Hornwort sporophytes grow from a persistent basal meristem, moss sporophytes grow initially from an apical initial, and subsequently from an intercalary meristem; the mechanism of proliferative growth in liverwort sporophytes appears to be intercalary meristem-like. *Selaginella* (lycophyte) and *Ceratopteris* (monilophyte) apices usually have two and one apical initials, respectively, but other lycophytes and monilophytes have apices with multiple initials. Spermatophyte (seed plant) apices are multicellular and functionally more complex. Figure adapted from Harrison (2017).

All land plants share a number of morphological traits—they are multicellular organisms with life cycles that alternate between a haploid gametophyte and a diploid sporophyte stage. Multicellular sporophytes develop within the maternal gametophyte (hence the name embryophytes for land plants) and produce sporopollenin-coated meiospores (Niklas and Kutschera, 2010). Extant members of the non-vascular bryophyte clade (i.e. mosses, liverworts, and hornworts; Figure 1) grow predominantly as haploid gametophytes. Following fertilization, bryophyte sporophytes are dependent on the parent gametophyte for nourishment and persist only briefly before undergoing meiosis and spore production. On the other hand in vascular plants (tracheophytes), the sister lineage to bryophytes consisting of lycophyte, monilophyte, and seed plant clades (Figure 1), the sporophyte stage is dominant and largely independent of the gametophyte (Niklas and Kutschera, 2010).

The phylogenetic relationship between the bryophytes and the tracheophytes has long been contentious, with almost every bryophyte grouping proposed to be sister lineage to the tracheophytes at some stage (Donoghue et al., 2021). However, wider taxon sampling combined with improved models of sequence evolution now strongly supports bryophytes as monophyletic, with liverworts and mosses forming a clade (the setaphytes) within the bryophytes (Figure 1; Wickett et al., 2014; Puttick et al., 2018; Sousa et al., 2019; Harris et al., 2020; Sousa et al., 2020). Likewise, improved phylogenetic methods have led to a reassessment of which group of the charophyte grade of green algae is the sister lineage to embryophytes. As they exhibit a number of traits characteristic of land plants (e.g. cell walls with plasmodesmata, asymmetric cell division, branching, and zygotic sporopollenin deposition), the Charophyceae and Coleochaetophyceae have historically been considered the likeliest closest embryophyte relatives (Mishler and Churchill, 1984; Mishler and Churchill, 1985). However, phylogenetic analyses of both organelle and nuclear sequences now support the aflagellate and predominantly unicellular Zygnematophyceae as sister lineage to the land plants (Turmel et al., 2006, 2013; Karol et al., 2010; Lemieux et al., 2016; Harris et al., 2020).

A true embryophyte phylogeny is critical to understanding how plants colonized land (Puttick et al., 2018; Donoghue et al., 2021). With the rejection of bryophytes as a paraphyletic grade, it is imperative to reassess previous conclusions about the ancestral state of land plants based on individual bryophyte lineages as sister to tracheophytes. Knowing the phylogenetic relationships between different land plants makes it possible to define which character traits are ancestral, which are derived, and which have been lost in specific lineages. Considering these different evolutionary trajectories, we can now more accurately infer the likely bodyplan, genetic architecture, and growth habit of the ancestral land plant. In this update, we focus on recent results from developmental genetic studies in non-seed plants to make inferences about how shoot apical growth was regulated in the ancestor of embryophytes.

## Structure of land plant shoot meristems

Apical growth (i.e. directional proliferative growth facilitated by the self-renewing activity of an undifferentiated meristematic cell or cells) was fundamental to the successful colonization of land as it enabled plants to spread across a solid substrate and, eventually, rise above it. The charophyte sister lineages to the land plants exhibit varied forms of apical growth, including filamentous and thallose habits reminiscent of extant bryophytes. For example, a single apical cell divides transversely in *Chara* to elongate the main filament, which is further extended by expansion of apical cell derivatives (Pickett-Heaps, 1967), in a mechanism similar to protonemal tip growth in moss (see below). Thus, apical growth evolved prior to the emergence of land plants and aspects of an ancestral body plan were retained following the colonization of land (Delwiche and Cooper, 2015; Harrison, 2017; Moody, 2020).

### Mosses

Following spore germination, moss gametophyte growth begins as a 2D filamentous network (the protonema) (Cove, 2005). Initially the protonema consists exclusively of chloroplast-rich chloronemal filaments. Depending on the species and the environment, filaments of longer and narrower caulonemal cells, with fewer chloroplasts, may also emerge. In both cases, filaments extend through tip growth and the uniplanar division of single apical stem cells (Menand et al., 2007). New apical cells initiate from cells subtending the tips of existing filaments (Cove and Knight, 1993; Kofuji and Hasebe, 2014). These side branch initials can either give rise to secondary filaments, therefore elaborating the 2D protonemal network or facilitate a transition to 3D growth through the formation of shoots (gametophores). Despite often initiating on the same parent cell, secondary filaments and gametophores can be distinguished from the orientation of the first cell division and the width of the initial cell (perpendicular/narrow and oblique/wide, respectively) (Harrison et al., 2009; Tang et al., 2020). Gametophore initials undergo three more rounds of cell division to generate a tetrahedral shaped apical cell, which subsequently cleaves spirally to produce a leafy shoot (Harrison et al., 2009). Although protonemal and gametophore apical cells share certain molecular signatures associated with cell proliferation, the multiplanar cell division pattern of gametophore apical cells is reflected in increased transcriptomic complexity (Frank and Scanlon, 2015b).

Moss reproductive organs (female archegonia and male antheridia) derive from single stem cells that initiate at gametophore tips and undergo predictable patterns of cell division, leading to gamete production (Landberg et al., 2013). After fertilization, moss sporophyte growth is characterized by limited rounds of division from an apical stem cell followed by proliferation of a more basal intercalary meristem (Figure 1; Sakakibara et al., 2008; Coudert et al., 2019). The duration and timing of intercalary meristem division is associated with the dramatically different sizes of moss sporophytes observed in different species (French and Paolillo, 1975a, 1975b). A recent transcriptomic study of *Physcomitrium patens* (sporophyte size ∼1 mm) and *Funaria hygrometrica* (sporophyte size ∼5 cm) suggests that size differences are largely a consequence of temporal shifts in the expression patterns of conserved genetic networks, rather than species-specific gene gain/loss (Kirbis et al., 2020).

### Liverworts

Relative to mosses, liverworts such as the model system *Marchantia polymorpha* display only a brief filamentous growth phase following spore germination (Nishihama et al., 2015). Subsequently, liverworts grow in either leafy or thallose forms. In the case of leafy forms, dichotomizing gametophores with ranked leaves (or phyllids) are generated by a single apical cell. In the case of thallose species such as *Marchantia*, thalli are iterated by the activity of a single wedge-shaped apical cell that resides in an apical notch. *Marchantia* apical cells divide in four planes, facilitating the formation of multiple cell layers within the thallus and a bifurcating pattern of thallus branching (Shimamura, 2016; Solly et al., 2017). Reproductive organs emerge vertically from the vegetative thallus on separate dioecious plants and, as in mosses, the developing sporophyte is nourished within the archegonia. However, unlike in moss sporophytes, there is no apical stem cell in the *Marchantia* sporophyte and proliferative growth appears limited to an intercalary meristem-like mechanism (Kienitz-Gerloff, 1874; Dierschke et al., 2021).

### Hornworts

Hornworts such as *Anthoceros agrestis* also grow vegetatively as thalli (Frangedakis et al., 2021). Similar to liverworts, growth is maintained by the activity of individual wedge-shaped apical cells that divide in four planes and are situated in apical notches (Renzaglia et al., 2008). However, apical notches form in greater numbers in *Anthoceros* and, consequently, thallus growth is irregular. Uniquely within the bryophytes, hornwort reproductive organs are embedded within the thallus. Furthermore, the sporophyte extends from a basal proliferative zone that remains active throughout its life cycle and spore production is continuous (Renzaglia et al., 2008). This is in sharp contrast to setaphytes, in which sporophyte proliferative growth is limited and spore production is terminal.

### Tracheophytes

In contrast to bryophytes, vascular plant apical growth occurs almost exclusively in the sporophyte generation, although apical initials have been retained in some fern gametophytes (Banks, 1999). The structure of vascular plant shoot meristems has been reviewed extensively elsewhere (e.g. Gifford and Corson, 1971; Steeves and Sussex, 1989; White and Turner, 1995; Barton, 2010; Spencer et al., 2021). To summarize briefly, in the lycophyte lineage there is considerable variation in shoot meristem organization. For example, in the Selaginellaceae, there are one or two transiently acting apical initials (Harrison et al., 2007), whereas in the Isoëtaceae and Lycopodiaceae, meristems contain multiple apical initials (Philipson, 1990). Monilophytes, including the model systems *Ceratopteris*, *Equisetum*, and *Nephrolepis* (Sanders et al., 2011; Frank and Scanlon, 2015a; Plackett et al., 2015), predominantly exhibit single apical cells; however, ferns with multiple apical cells have also been reported (White and Turner, 1995). Proliferative “core” groups of cells that subtend apical initials and have unique transcriptional signatures have been identified in lycophytes and ferns, suggesting that apical growth in these lineages is coordinated by multiple cell types (Frank et al., 2015; Ambrose and Vasco, 2016).

Shoot apical meristems (SAMs) in seed plants are larger and more complex, consisting of multiple stem cells and distinct functional zones. For example, in flowering plants such as Arabidopsis (*Arabidopsis thaliana*), meristems consist of three domains: the central (CZ), peripheral (PZ) and rib (RZ) zones. Cells within the CZ self-renew and replenish the surrounding (PZ) and subtending (RZ) zones and are therefore considered the stem cells (functionally equivalent to the apical cells of non-seed plants). Cells within the PZ and RZ proliferate more rapidly than in the CZ and ultimately differentiate to produce lateral organs and ground tissue, respectively (Steeves and Sussex, 1989; Barton, 2010).

It is possible that the ancestral land plant had a multicellular meristem which was subsequently reduced in the bryophyte lineage. However, the similarity of (1) charophyte and bryophyte patterns of growth from single apical initials and (2) extant moss sporophytes and fossils of proto-tracheophytes with apparently simple apical domains (Harrison and Morris, 2017) suggest that meristem complexity is a derived trait in vascular plants. The polyphyletic distribution of multicellular apices within the tracheophytes (i.e. within specific lycophyte and monilophyte lineages), and unique transcriptomic signatures of lycophyte and monilophyte apical cells (Frank et al., 2015), suggest sporophytic meristems may have evolved convergently. How indeterminate vascular plant meristems evolved from an ancestor with a diminutive and determinate sporophyte remains a critical question in the field. The vascular plant SAM may have evolved from persistence and expansion of a transient moss-like sporophyte apical initial (Albert, 1999). Alternatively, a bryophyte-type sporophyte intercalary meristem may have moved apically during evolution and be homologous to SAM proliferative zones (e.g. the core cells of lycophytes and ferns, and PZ/RZ of angiosperms) (Mishler and Churchill, 1984). It is also possible that both these scenarios occurred and the vascular plant SAM evolved from a juxtaposition of the apical and intercalary domains (Harrison, 2017).

It has been proposed that the elaboration of sporophytic apical growth required the large-scale co-option of gametophytic genetic networks (Floyd and Bowman, 2007; Niklas and Kutschera, 2010). Supporting this idea, transcriptomic studies have found homologs of multiple angiosperm SAM regulators to be expressed in *Physcomitrium* gametophytes (Nishiyama et al., 2003; Frank and Scanlon, 2015a). However, the observation that a number of critical sporophyte stem cell regulators do not function in bryophyte gametophyte apical growth suggests this is not universally true (Sakakibara et al., 2008; Sakakibara et al., 2014; Yip et al., 2016). In the following sections, we review molecular studies into bryophyte development and discuss the likely ancestral roles for regulators of land plant shoot development.

## Genetic networks that control apical growth

### *CLAVATA*/*WUSCHEL-LIKE HOMEOBOX*

Variants of the *CLAVATA* (*CLV*)–*WUSCHEL* (*WUS*) genetic network are essential regulators of stem cell proliferation in a variety of angiosperm meristematic contexts. In the Arabidopsis SAM (for details of *CLV* signaling in other developmental contexts, see Fletcher, 2020), the small signaling peptide CLV3 is expressed in the outer cell layers of the CZ (Fletcher et al., 1999). The peptide migrates basally to the center of the SAM where it is bound by its principal receptor—the leucine-rich repeat receptor like kinase (LRR-RLK) CLV1 (Clark et al., 1997; Brand et al., 2000). On the flanks of the SAM, CLV1 functions redundantly with the closely related BARELY ANY MERISTEM (BAM) receptors (Nimchuk et al., 2015). CLV1/BAM receptors, in combination with additional LRR-RLK family members CLV2 (Kayes and Clark, 1998), RECEPTOR-LIKE PROTEIN KINASE 2 (RPK2) (Kinoshita et al., 2010), and CLAVATA3-INSENSITIVE RECEPTOR KINASES (CIKs) (Hu et al., 2018), plus the presumptive pseudokinase CORYNE (CRN) (Müller et al., 2008), function to transcriptionally repress the transcription factor WUS. WUS promotes stem cell proliferation and so, in effect, CLV3 and its downstream signaling components act to limit stem cell number. To maintain stem cell homeostasis, WUS moves apically through plasmodesmata to promote *CLV3* expression, thus completing a negative feedback loop (Brand et al., 2000; Schoof et al., 2000). Recently the CLV3-like small peptide CLE40, which is expressed in the PZ and bound by BAM1, has been shown to promote *WUS* activity (Schlegel et al., 2021). CLV-mediated regulatory networks therefore buffer SAM stem cell number through positive and negative relationships with *WUS*.

Orthologs of *CLV3*, *CLV1/BAM1*, *RPK2*, and *CIK* are present in bryophyte genomes, whereas *CLV2* and *CRN* emerged in vascular plants. None of these genes are present in charophyte algae, suggesting that a core *CLV* signaling network evolved in the ancestor of land plants and was later elaborated during land plant evolution (Whitewoods et al., 2018; Furumizu et al., 2021; Takahashi et al., 2021). In *Physcomitrium*, loss-of-function *Ppclv1a/1b* and *Pprpk2* mutants produce an over-proliferation of apical cells at the base of gametophores. Moreover, application of synthetic CLV3-like peptides inhibits cell proliferation and causes gametophore dwarfing (Whitewoods et al., 2018). In *Marchantia*, a CLV3–CLV1–CIK module also regulates gametophytic stem cell proliferation, although in this case positively (Hirakawa et al., 2020; Takahashi et al., 2021). Intriguingly, a *CLV3* family member that promotes stem cell proliferation in Arabidopsis has been found to repress apical growth in *Marchantia* (Hirakawa et al., 2019), suggesting a divergence of signaling mechanisms during evolution. Together, these results support the idea that *CLV3* functioned to regulate stem cell proliferation in the common ancestor of land plants. Whether or not *CLV* signaling was co-opted for sporophytic apical growth in vascular plants, or was already functional in the ancestral sporophyte, will require closer analysis of bryophyte *clv* mutant sporophytes.

In addition to a role in stem cell proliferation, *CLV* signaling also appears to regulate stem cell identity in bryophytes. Reduced *CLV* signaling in *Physcomitrium*, most notably in *Pprpk2* mutants, enhances protonemal spread and the chloronema–caulonemal transition. These observations, and the strong expression of *CLV* signaling components in protonemal tips, suggest that *CLV* signaling represses caulonemal apical cell identity (Nemec Venza et al., 2022). A role for the *CLV* pathway in moss gametophore initiation has also been identified (Whitewoods et al., 2018). *Ppclv* receptor mutants, and artificial miRNA-targeting of *CLV3* homologs, inhibit gametophore formation—in this case not through a defect in stem cell identity specification, but rather through perturbed cell division plane orientation following bud initiation (Whitewoods et al., 2018). *CLV* signaling also regulates the angle of cell division in *Marchantia* and Arabidopsis (Whitewoods et al., 2018; Hirakawa et al., 2019), suggesting that this may be a further ancestral function.

With regard to the architecture of the ancestral *CLV* genetic network, the identity of the downstream targets remains a critical unknown. In angiosperms, *CLV* signaling is mediated largely by members of the *WUS* clade of *WUS-LIKE HOMEOBOX* (*WOX*) genes. However, the *WUS* clade of *WOX* genes emerged after the split between bryophytes and tracheophytes, loss-of-function *Ppwox13la/b* mutants do not affect *Physcomitrium* gametophore development, and *MpWOX* is not required for *CLV3* signaling in *Marchantia* (Nardmann and Werr, 2012; Sakakibara et al., 2014; Hirakawa et al., 2020). These results strongly suggest that the *CLV–WOX* regulatory interaction is derived. What, then, was the ancestral target(s) of the *CLV* network? Recent work points to plant hormones as likely candidates. In *Physcomitrium*, the *Pprpk2* and *Ppclv1a/1b* protonema phenotypes are dependent on auxin transport and it has been proposed that *CLV* signaling regulates the flow of auxin in protonema tip cells (Nemec Venza et al., 2022). In Arabidopsis, the effects of CLV2/CRN (which are tracheophyte-specific and CLV1-independent) on flower development are auxin-mediated (Jones et al., 2021), suggesting that regulatory interactions between *CLV* signaling components and auxin have evolved repeatedly in land plants.

Using the observed effects of cytokinin treatments on *Ppclv1a/1b* and *Pprpk2* as inputs for mathematical models, it has also been suggested that *PpCLV1A/1B* function upstream of a cytokinin signaling pathway (Cammarata et al., 2022). Because *WUS* is known to mediate both auxin and cytokinin signaling in angiosperms (Leibfried et al., 2005; Ma et al., 2019), these findings support an exciting model in which *WUS* was co-opted to act as an intermediate between *CLV* and hormone pathways during vascular plant evolution. As *WUS*-like gene activity appears limited to the vasculature and roots of *Selaginella* and *Ceratopteris*, a role for the *WUS* clade in shoot apical growth appears derived within the vascular plants (Youngstrom et al., 2022).

In bryophytes, following presumed extensive gene loss, members of the *WOX* family are restricted to the T1 clade that includes homologs of *WOX13* (Nardmann and Werr, 2012; Wu et al., 2019). *Physcomitrium PpWOX13LA/B* are required for embryonic cell division and the de novo formation of stem cells (Sakakibara et al., 2014) and *PpWOX13LB* is able to rescue callus production defects in the Arabidopsis *wus13* mutant (Ikeuchi et al., 2022). Thus, *WOX* genes appear to function ancestrally both within the diploid generation and in the regulation of stem cell activity. The smaller thalli observed in *Marchantia Mpwox* mutants (Hirakawa et al., 2020), and effects on both generations of *WOX* knockdown in a fern (Youngstrom et al., 2019), suggest that *WOX* genes regulated cell proliferation in both stages of the ancestral land plant life cycle.

### HAIRY MERISTEM

The *HAIRY MERISTEM* (*HAM*) clade of GRAS family transcription factors are critical regulators of shoot indeterminacy in angiosperms, where they physically and genetically interact with WUS-type WOX proteins to promote stem cell proliferation (Engstrom et al., 2011; Zhou et al., 2015). *HAM* genes evolved prior to the emergence of land plants (although have since been lost in *Marchantia*) and have been sufficiently conserved during evolution that moss, lycophyte, and fern *HAM* orthologs are able to rescue an Arabidopsis *ham* mutant (Geng et al., 2021). Conservation of function is further supported by a genetic analysis of the *Physcomitrium HAM* gene *PpGRAS12*. Loss of *PpGRAS12* activity through gene targeting restricts gametophore outgrowth whereas overexpression promotes the formation of ectopic apical cells (Beheshti et al., 2021). This suggests that *HAM* genes promoted stem cell proliferation in the ancestral land plant and, as with *CLV*, that their regulatory interaction with *WOX* genes is derived.

### KNOTTED-LIKE HOMEOBOX

Members of the three amino acid loop extension (TALE) superfamily of homeobox transcription factors are essential for apical growth (Hay and Tsiantis, 2010). There are two families of TALE genes in plants: *KNOTTED-LIKE HOMEOBOX* (*KNOX*) and *BELL-LIKE* (*BELL*). *KNOX* genes can be subdivided into two classes and members of both classes heterodimerize with BELL partners to regulate development. In angiosperms, Class I and Class II *KNOX* genes act antagonistically: Class I genes promote meristematic activity and stem cell identity whereas Class II genes promote differentiation (Vollbrecht et al., 1991; Long et al., 1996; Furumizu et al., 2015).

The duplication that generated Class I and Class II *KNOX* genes occurred in charophyte algae (Frangedakis et al., 2017). In the unicellular chlorophyte *Chlamydomonas reinhardtii*, a single KNOX and BELL protein are each expressed in distinct gametes, but heterodimerize and activate the zygotic genetic program after gamete fusion (Lee et al., 2008). Thus, the initiation of the diploid genetic program appears to be the ancestral function of *KNOX/BELL* genes (Bowman et al., 2016). This role has been maintained in bryophytes. Similar to *Chlamydomonas*, expression of Class I *KNOX* and *BELL* genes in gametes is required to establish zygotic development in *Marchantia* (Dierschke et al., 2021; Hisanaga et al., 2021). *Physcomitrium* Class II *KNOX* genes repress gametophytic development during early sporophytic growth (Sakakibara et al., 2013) and *PpBELL1* over-expression is able to ectopically induce the diploid program during the gametophytic phase (Horst et al., 2016).

Analyses of moss Class I *KNOX* genes suggest that their role in sporophyte meristematic growth arose in the ancestral land plant. Loss-of-function Class I *KNOX Physcomitrium* mutants have no effect on gametophytic growth but sporophyte development is stunted due to reduced proliferation of the intercalary meristem (Singer and Ashton, 2007; Sakakibara et al., 2008; Coudert et al., 2019). In angiosperms, Class I *KNOX* genes promote stem cell identity via cytokinin signaling (Jasinski et al., 2005; Yanai et al., 2005). This functional interaction also appears to have evolved in the ancestral land plant as *Physcomitrium* Class I *KNOX* activity is at least in part dependent on cytokinin (Coudert et al., 2019). The meristematic expression of Class I *KNOX* genes in diverse land plant lineages (Harrison et al., 2005; Sano et al., 2005; Ambrose and Vasco, 2016; Dierschke et al., 2021), and their broad functional conservation (Frangedakis et al., 2017) suggests that the recruitment of *KNOX* activity was fundamental to the evolution of indeterminate sporophyte growth.

### Class III HD-ZIP

The absence of an obvious gametophytic role for bryophyte Class I *KNOX* genes—critical regulators of sporophyte meristem development—challenges the hypothesis that indeterminate vascular shoot growth required widescale redeployment of gametophytic apical networks. The view that sporophyte elaboration did not require widescale gametophytic network co-option is supported by a study of Class III HD-ZIP (C3HDZ) genes in *Physcomitrium.* C3HDZ genes, which arose in the charophytes (Floyd et al., 2006), are expressed in divergent tracheophyte apices (Floyd et al., 2006; Prigge and Clark, 2006) and promote SAM formation in angiosperms (McConnell et al., 2001; Emery et al., 2003). However, loss of C3HDZ function has no effect on apical growth in the gametophore (Yip et al., 2016). Moreover, strong C3HDZ expression in the transient sporophyte apical initial could indicate a pre-existing sporophytic role.

### LEAFY

The plant-specific transcription factor LEAFY (LFY) principally regulates meristem identity during reproductive development in angiosperms (Weigel et al., 1992). In *Physcomitrium*, two *LFY* genes regulate the first zygotic division and cell divisions at later stages of sporophytic development (Tanahashi et al., 2005). As moss and hornwort LFY proteins bind lineage-specific motifs, whereas liverwort and vascular plant LFY bind a conserved motif, and *PpLFY* is not able to rescue the Arabidopsis *lfy* mutant phenotype, it is unclear whether the role of *PpLFY* is reflective of an ancestral state, or divergent downstream networks (Maizel et al., 2005; Sayou et al., 2014). Gene expression (Rodríguez-Pelayo et al., 2022) and functional (Plackett et al., 2018) analyses in ferns and a lycophyte support an ancestral role for *LFY* in early embryo development. However, the results of these studies further suggest that *LFY* was co-opted to regulate cell division in wide-ranging developmental contexts during vascular plant diversification.

### TEOSINTE BRANCHED 1

Based on the phylogenetic distribution of branching, it has been proposed that sporophyte branching evolved after the split between the bryophyte and tracheophyte lineages (Harrison, 2017). In angiosperms, shoot branching is regulated by multiple pathways, including by Class II *TEOSINTE BRANCHED1/CYCLOIDEA/PROLIFERATING CELL FACTOR1 (TCP)* genes, which repress the outgrowth of axillary meristems (Doebley et al., 1997; Aguilar-Martínez et al., 2007; Koyama et al., 2007). A transcriptomic analysis in moss has revealed that expression of the two *Physcomitrium* Class II *TCP* genes (*PpTCP5/6*) is enriched in the sporophyte and, intriguingly, that loss of *PpTCP5* function induces sporophytic branching (Ortiz-Ramírez et al., 2016). Class II *TCP* genes are thus likely ancestral repressors of sporophyte branching in land plants. Moss sporophyte branching is also observed in plants with perturbed auxin transport (Fujita et al., 2008; Bennett et al., 2014b). In combination, these results suggest that the sporophyte of the ancestral land plant was competent to branch. However, at least in extant lineages, bryophyte branching was either lost, or restricted by conserved developmental networks that were reconfigured during tracheophyte evolution.

### AINTEGUMENTA-LIKE

Members of the *AINTEGUMENTA* (*ANT*) subfamily of AP2-LIKE transcription factors regulate a variety of processes in angiosperms, including stem cell maintenance, cell proliferation in young organs, and embryogenesis (Elliott et al., 1996; Boutilier et al., 2002; Galinha et al., 2007). A reverse genetic study in *Physcomitrium* found that four *ANT* family members (*APB1-4*) redundantly regulate gametophore development (Aoyama et al., 2012). *APB1-4* function downstream of auxin to specify the gametophore branch initial. Due to the expansion and functional diversity of the *ANT* gene family in angiosperms, it is difficult to surmise ancestral function but, with no report of an *Ppapb1-4* sporophyte phenotype (Aoyama et al., 2012), this perhaps represents an example of gametophytic apical network co-option.

### DEFECTIVE KERNEL 1

An additional class of genes that regulate gametophore formation is the *DEFECTIVE KERNEL 1* (*DEK1*) family of calpains. Angiosperm *dek1* mutants are embryo lethal due to disrupted patterns of cell division (Johnson et al., 2005; Lid et al., 2005). The orientation of cell division appears to be the ancestral role for *DEK1* as *Physcomitrium Ppdek1* mutants are unable to correctly specify gametophore apical initial position (Perroud et al., 2014). Furthermore, *PpDEK1* is able to rescue Arabidopsis *dek1* mutant phenotypes (Liang et al., 2013). Based on network analyses of transcriptomic data, it has been inferred that PpDEK1, which is localized to the plasma membrane in recently divided cells (Perroud et al., 2020), functions as a developmental “gatekeeper” of cell fate transitions (Demko et al., 2021). In the case of the transition to 3D growth during gametophyte development, PpDEK1 appears to function partly through repression of *APB* genes (Demko et al., 2014).

### NO GAMETOPHORES

Further regulators of 3D apical growth have been identified by Moody and colleagues using elegant forward genetic screens that use somatic hybridization to facilitate bulk segregant analysis in polyploid *Physcomitrium* (Moody et al., 2018a). This strategy has revealed *NO GAMETOPHORES 1* (*NOG1*) and *NOG2* as critical determinants of gametophore initiation. *PpNOG1* encodes an ubiquitin-associated protein that functions upstream of *APB* genes to promote gametophore bud formation and, subsequently, orient apical cell divisions (Moody et al., 2018b). A lack of functional analyses for *NOG1* orthologs in other land plant lineages limits speculation regarding an ancestral function. However, as *NOG1* genes are only found in land plants, it has been proposed that *NOG1* evolution was fundamental to the establishment of 3D growth (Moody et al., 2018b). Like *NOG1*, *CLV* pathway genes are also critical regulators of apical cell division plane orientation that emerged in the ancestor of land plants, and both regulate rotating division planes of apical cells. As rotation of apical cell division plane was a critical land plant innovation that facilitated 3D growth (Zimmerman, 1952), the relative timing, and potential interdependence, of their evolution is a fascinating open question.

*PpNOG2*, which encodes a shikimate o-hydroxycinnamoyltransferase that functions in the ascorbic acid pathway, restricts gametophore initial cell formation but is required for apical growth of the emerging bud (Moody et al., 2021). Auxin homeostasis and *CLV* genes are misregulated in *Ppnog2*, which is proposed to act downstream of NOG1/DEK1/APBs. Inhibition of the Arabidopsis NOG2 ortholog HCT can perturb the ascorbic acid pathway, disrupting flavonoid biosynthesis and consequently limiting auxin transport (Besseau et al., 2007). Thus, the regulation of auxin homeostasis was likely an ancestral function for *NOG2.*

### LATERAL ORGAN SUPRESSOR 1

Demarcation between self-renewing meristematic cells and cells differentiating into lateral organs is critical to apical growth. A number of genes are expressed at the boundary between lateral organs and the SAM in angiosperms, including members of the *ALOG* family (Hepworth and Pautot, 2015). In addition to regulating lateral organ development cell autonomously, angiosperm *ALOG* genes also regulate meristem development non-cell autonomously (Takeda et al., 2011; MacAlister et al., 2012; Yoshida et al., 2013). A recent study of the *Marchantia ALOG* gene *LATERAL ORGAN SUPRESSOR 1* (*LOS1*) suggests both these functions are ancestral in land plants. Expression of *MpLOS1* is restricted to lateral organs, however, *Mplos1* mutants produce aberrant lateral organs and fail to maintain an apical meristem (Naramoto et al., 2019). Mobile signaling between meristematic and differentiating cells therefore appears to be a fundamental feature of apical growth.

## Hormonal coordination of apical growth

### Auxin

Many of the genetic modules outlined above are integrated into wider hormonal signaling pathways, not least the auxin network (e.g. Aoyama et al., 2012; Moody et al., 2021; Nemec Venza et al., 2022). Auxin regulates angiosperm development in a myriad of ways. Specifically in regard to shoot apical growth, auxin promotes the differentiation of lateral organs on the periphery of the SAM whereas stem cell activity requires a minimal level of auxin signaling (Ma et al., 2019). Moreover, basipetal transport of auxin from the primary SAM inhibits shoot branching, predominantly through repression of axillary meristem outgrowth (Prusinkiewicz et al., 2009). A role for auxin in meristem maintenance and polar auxin flow from the SAM are conserved across the tracheophytes (Sanders and Langdale, 2013).

Perturbation to auxin transport and signaling has shown that auxin also controls the balance between apical growth and differentiation in bryophytes. Furthermore, auxin signaling is necessary in both gametophytic and sporophytic stages of bryophyte life cycles (Ishizaki et al., 2012; Bennett et al., 2014b; Viaene et al., 2014; Flores-Sandoval et al., 2015; Kato et al., 2015). Charophyte algae have orthologs of many auxin signaling pathway components in their genomes and exhibit a capacity for long distance polar auxin transport (De Smet et al., 2011; Boot et al., 2012). The core molecular components of auxin transport, perception, signaling, and biosynthesis are all found in bryophyte genomes (Paponov et al., 2009; Eklund et al., 2010; Prigge et al., 2010; Bennett et al., 2014a; Flores-Sandoval et al., 2015; Kato et al., 2015; Lavy et al., 2016; Plavskin et al., 2016; Li et al., 2020; Zhang et al., 2020). Together, these results suggest that a basic auxin response module was present in the ancestral land plant and that this module regulated apical growth in the ancestral gametophyte and sporophyte.

### Cytokinin

Cytokinin has long been known to promote shoot apical activity cell throughout the land plant lineage (Skoog and Miller, 1957; Ashton et al., 1979; Reski and Abel, 1985). In angiosperms, as referenced above, cytokinin signaling is a critical effector of the *WUS* and *KNOX* meristem maintenance pathways. Through conserved genetic networks, cytokinin induces gametophore apical initiation formation in *Physcomitrium* and regulates multiple aspects of *Marchantia* thallus development (Flores-Sandoval et al., 2016; von Schwartzenberg et al., 2016; Aki et al., 2019; Hyoung et al., 2020). Importantly, cytokinin also promotes meristematic proliferation in moss sporophytes (French and Paolillo, 1975b; Coudert et al., 2019), suggesting that a role for cytokinin in sporophyte development evolved in the ancestral land plant.

## Concluding remarks

The results of the studies summarized above present a complex picture of how apical growth evolved in land plants (Table 1). Together, the findings support models of tracheophyte sporophyte co-option of gametophytic networks (e.g. *CLV*, *HAM*, *APBs*, and *ALOG*), independent recruitment of ancestral sporophyte networks (e.g. *LFY*) and the conservation of ancestral sporophyte function (e.g. Class I *KNOX*, Class II *TCP*).

**Table 1:** Summary of functional studies into apical growth in bryophytes

Gene family	Function in bryophytes	Function in vascular plant SAMs (sporophytes)	Putative ancestral function
*CLV3/CLV1/RPK2*	To regulate cell proliferation, cell identity, and cell division planes in gametophyte (*Physcomitrium*, *Marchantia*)	To regulate cell proliferation, cell identity, and cell division planes (angiosperms)	To regulate cell proliferation, cell identity, and cell division planes
*WOX*	To promote cell division in the zygote (*Physcomitrium*) and gametophyte (*Marchantia*)	To promote cell proliferation (vascular plants)	To promote cell proliferation
*HAM*	To promote stem cell proliferation in the gametophyte (*Physcomitrium*)	To promote stem cell proliferation (angiosperms)	To promote stem cell proliferation
*KNOX*	Class I: to promote sporophyte meristematic growth (*Physcomitrium*) and the transition to the sporophyte stage (*Marchantia*) Class II: to repress the gametophyte stage (*Physcomitrium*)	Class I: to promote meristematic growth (vascular plants) Class II: to promote differentiation, in part through antagonizing Class I function (angiosperms)	Pre-duplication: to promote the diploid genetic program and life cycle progression Class I: to promote sporophyte meristematic growth Class II: ?
*C3HDZ*	To promote cell proliferation and regulate tissue patterning in gametophyte leaf development (*Physcomitrium*)	To promote meristem maintenance and pattern shoots and lateral organs (vascular plants)	Domain specification in lateral organs?
*LFY*	To regulate cell division in the embryo and sporophyte (*Physcomitrium*)	To promote shoot indeterminacy (*Ceratopteris*) To promote reproductive development (vascular plants) To maintain indeterminate cell fate in branches (angiosperms)	To regulate sporophytic cell division To promote sporangium formation?
*Class II TCP*	To repress sporophyte branching (*Physcomitrium*)	To repress sporophyte branching (angiosperms)	To repress sporophyte branching
*APBs*	To specify the gametophore apical initial (*Physcomitrium*)	To promote stem cell and young tissue proliferation (angiosperms)	To promote stem cell identity
*DEK1*	To orient gametophore apical cell division (*Physcomitrium*)	To orient cell division planes during embryogenesis (angiosperms)	To orient cell division planes
*NOG1*	To promote gametophore initial identity (*Physcomitrium*)	?	?
*NOG2*	To regulate gametophore formation and auxin homeostasis (*Physcomitrium*)	To regulate flavonoid biosynthesis (angiosperms)	To regulate auxin homeostasis via flavonoid levels
*ALOG*	Non-cell autonomous regulation of meristem maintenance (*Marchantia*)	Non-cell autonomous regulation of meristem maintenance (angiosperms)	Non-cell autonomous regulation of meristem maintenance

*Notes*: The lineage that reverse genetic studies were carried out in is indicated in parentheses (“angiosperms” and “vascular plants” indicate genetic evidence from divergent lineages within these clades). Putative ancestral function is predicted based on conservation between bryophyte and tracheophyte lineages, with sporophyte-specific roles indicated where supported. References can be found in main text.

With functional genetic data from a highly limited subset of land plant lineages, our assignations of ancestral roles are somewhat speculative and depend on assumptions that may shift with further investigation. In particular, they assume a model of land plant evolution in which similar gene function in bryophytes and tracheophytes reflects a common evolutionary history. It is also possible that a superficially conserved function reflects parallel evolution of a core genetic network. Distinguishing between these evolutionary trajectories is challenging and requires character mapping in additional land plant lineages (see the “Outstanding Questions”). This problem is compounded by high rates of gene loss and morphological simplification within the bryophytes (Harris et al., 2020; Donoghue et al., 2021). The recent strong support for the reclassification of bryophytes as a monophyletic group, however, strengthens the inferences we can now make about ancestral function.

**Figure 2:**
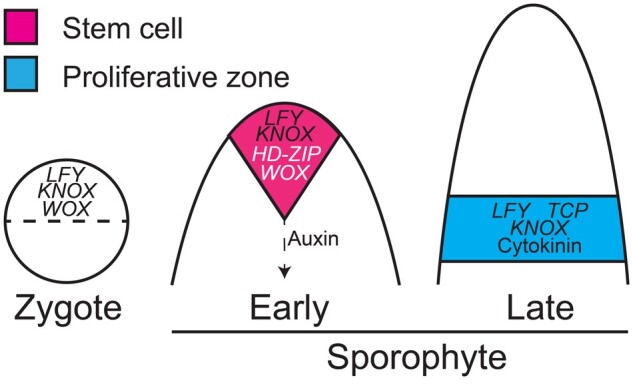
Putative apical regulators in the ancestral sporophyte. Hypothetical ancestral sporophyte based on extant *Physcomitrium* morphology. Putative roles inferred from bryophyte gene function that are broadly conserved in vascular plants. In zygotes, *WOX* and *LFY* genes are required for the first cell division and *KNOX* genes promote establishment of the sporophyte (Tanahashi et al., 2005; Sakakibara et al., 2013, 2014; Dierschke et al., 2021). In sporophytes, *PpLFY* genes are expressed throughout development and *Pplfy* mutants have disrupted cell division planes in apical and basal regions (Tanahashi et al., 2005); Class I *KNOX* genes regulate cell division in the apical initial and intercalary meristem, their role in the intercalary meristem is dependent on cytokinin signaling (Sakakibara et al., 2008; Coudert et al., 2019); putative roles for *HD-ZIP III* and *WOX* genes in apical initial proliferation are based on gene expression patterns rather than functional data (indicated by white lettering) (Sakakibara et al., 2014; Yip et al., 2016); maintenance of an auxin minimum in the apical initial by basipetal auxin transport promotes stem cell identity (Fujita et al., 2008; Bennett et al., 2014b; Thelander et al., 2019; Nemec Venza et al., 2022); Class II *TCP* genes repress branching in the proliferative zone (Ortiz-Ramírez et al., 2016).

Further characterization of bryophyte development will also improve our understanding of ancestral genetic networks. Specifically, additional functional studies of sporophyte development will enable us to determine whether gene families were functional in the ancestral sporophyte, or have been co-opted from gametophytic apical networks. For example, current data suggest that the *CLV* and *APB* networks were co-opted from roles in gametophytic development to regulate apical growth in tracheophyte sporophytes (Aoyama et al., 2012; Whitewoods et al., 2018). More detailed analyses of sporophyte development in *Ppclv* and *Ppapb* mutants may reveal ancestral sporophytic roles. The relative intractability of model bryophyte sporophytes makes such experiments difficult. However, recent results demonstrate how important investigating sporophyte development is for elucidating how plants first grew on land. Thanks to a combination of developmental genetic (e.g. Tanahashi et al., 2005; Bennett et al., 2014b; Sakakibara et al., 2014; Ortiz-Ramírez et al., 2016; Coudert et al., 2019) and phylogenetic (e.g. Puttick et al., 2018) studies we now understand the ancestral sporophyte to be far more complex than first envisioned (Figure 2). With a wider diversity of land plant lineages being adopted as model systems (e.g. Plackett et al., 2015; Uddenberg et al., 2015; Conway and Di Stilio, 2020; Di Stilio and Ickert-Bond, 2021; Frangedakis et al., 2021; Spencer et al., 2021), and new fossil discoveries continuing to calibrate morphological trajectories (Edwards et al., 2022a, 2022b), further complexity is likely to be uncovered.

ADVANCES

- Improved phylogenetic modeling has reclassified the bryophytes and tracheophytes as sister lineages; this updated phylogeny has led to a reassessment of the nature of the ancestral land plant.
- Recent reverse genetic studies into bryophyte models have provided multiple insights into the evolution of core developmental networks in plants.
- The ancestral land plant had a sporophyte that was morphologically far more complex than previously predicted.
- Indeterminate growth in vascular plants was therefore likely more preconditioned by genetic networks in the ancestral sporophyte, and less dependent on gametophytic co-option, than previously thought.

OUTSTANDING QUESTIONS

- Do genes that regulate cell division in bryophyte zygotes (i.e. *WOX*, *LFY*) also regulate later stages of sporophyte development?
- Does the *CLV* pathway regulate bryophyte sporophyte development?
- How and when were *WOX* genes co-opted into the *CLV* signaling network?
- What is the function of Class II *KNOX* genes in bryophytes?
- How labile is the repression of branching in bryophyte sporophytes?
- How is proliferative growth regulated in the liverwort sporophyte?
- How conserved are core developmental genetic networks in hornworts?
- Are angiosperm SAM stem cells and proliferative zones homologous to the moss sporophyte apical cell and intercalary meristem, respectively?

## Notes

J.P.F.: writing—original draft preparation. C.J.H.: writing—reviewing and editing.

The author responsible for distribution of materials integral to the findings presented in this article in accordance with the policy described in the Instructions for Authors (https://academic.oup.com/plphys/pages/general-instructions) is Jim Fouracre (jim.fouracre@bristol.ac.uk).
